# 24-Epibrasinolide Modulates the Vase Life of Lisianthus Cut Flowers by Modulating ACC Oxidase Enzyme Activity and Physiological Responses

**DOI:** 10.3390/plants10050995

**Published:** 2021-05-17

**Authors:** Mohammad Darvish, Habib Shirzad, Mohammadreza Asghari, Parviz Noruzi, Abolfazl Alirezalu, Mirian Pateiro, Aseel Takshe, José Manuel Lorenzo

**Affiliations:** 1 Department of Horticultural Sciences, Faculty of Agriculture, Urmia University, Urmia 5756151818, Iran; mohammadadarvish731@gmail.com (M.D.); m.asghari@urmia.ac.ir (M.A.); p.noruzi@urmia.ac.ir (P.N.); a.alirezalu@urmia.ac.ir (A.A.); 2 Centro Tecnológico de la Carne de Galicia, Parque Tecnológico de Galicia, rúa Galicia n° 4, San Cibrao das Viñas, 32900 Ourense, Spain; mirianpateiro@ceteca.net; 3 Environmental Health Sciences, Faculty of Communication, Arts and Sciences, Canadian University Dubai, Dubai 117781, United Arab Emirates; aseel.takshe@cud.ac.ae; 4 Área de Tecnología de los Alimentos, Facultad de Ciencias de Ourense, Universidad de Vigo, 32004 Ourense, Spain

**Keywords:** lisianthus, ACC oxidase, ethylene, anthocyanins, malondialdehyde, vase life

## Abstract

Ethylene is the most important factor playing roles in senescence and deterioration of harvested crops including cut flowers. Brassinosteroids (BRs), as natural phytohormones, have been reported to differently modulate ethylene production and related senescence processes in different crops. This study was carried out to determine the effects of different levels of 24-epibrassinolide (EBL) on ACC oxidase enzyme activity, the final enzyme in ethylene biosynthesis pathway, vase life, and senescence rate in lisianthus cut flowers. Harvested flowers were treated with EBL (at 0, 3, 6, and 9 µmol/L) and kept at 25 °C for 15 days. The ACC oxidase activity, water absorption, malondialdehyde (MDA) production and vase solution absorption rates, chlorophyll and anthocyanin contents, and the vase life of the flowers were evaluated during and at the end of storage. EBL at 3 µmol/L significantly (*p* ≤ 0.01) enhanced the flower vase life by decreasing the ACC oxidase activity, MDA production and senescence rates, and enhancing chlorophyll and anthocyanin biosynthesis and accumulation, relative water content, and vase solution absorption rates. By increasing the concentration, EBL negatively affected the flower vase life and postharvest quality probably via enhancing the ACC oxidase enzyme activity and subsequent ethylene production. EBL at 6 and 9 µmol/L and in a concentration dependent manner, enhanced the ACC oxidase activity and MDA production rate and decreased chlorophyll and anthocyanin accumulation and water absorption rate. The results indicate that the effects of brassinosteroids on ethylene production and physiology of lisianthus cut flowers is highly dose dependent.

## 1. Introduction

Lisianthus (*Eustoma grandiflorum*), is an attractive and popular potted and cut flower that is becoming one of the most highly valued cut flowers in local and international markets [1]. Due to its rose-like flower shapes and a wide range of beautiful colors, it has become more famous in recent years so that it is now among the top 10 cut flowers in the world, and the market of this flower has increased to more than 50% over the past 10 years [1,2,3]. Flowers are fast living organs of the plants with a high metabolic activity making them very susceptible to postharvest losses [4]. For this reason, the vase life is a commercial attribute determining the flexibility of the market at any time and an important indicator for cut flowers value. Similar to most other cut flowers, lisianthus has a relatively short vase life and extending the vase life of the flower is a major concern for the producers [3,5].

A series of unwanted events including water loss, activity of deferent degrading enzymes, respiration, pathogens, and physiological disorders accelerate the senescence process in harvested crops including the cut flowers, resulting in the loss in quality and marketability [6]. The activity of free radicals and reactive oxygen species (ROS) produced due to normal cellular metabolism, and also the activity of different enzymes degrading cell walls, membranes, chlorophylls, and other cell organelles are the main factors enhancing the senescence rate [6]. Ethylene has been demonstrated to be the most important key signal playing role in plant cell and tissue senescence processes via activating the gene expression and enzyme activity of different degrading enzymes including lipoxygenases, lipases, pectinases, proteinases, polygalacturonases, and chlorophyllases [7,8,9]. The role of ethylene in enhancing the respiration rate resulting in an oxidative burst due to excess free radical and ROS production has been well demonstrated [4,10]. Furthermore, the enhanced respiration rate is the cause for carbohydrate depletion in harvested crops leading to accelerated senescence and breakdown [6]. This is of most importance in cut flowers, which are the active growing parts of the plants and have no further reserves for consumption during elevated respiration rates. Therefore, ethylene production and action is the most important factor affecting the postharvest life and quality of harvested perishable crops including the cut flowers [4].

The phytohormones and plant growth regulators (PGRs) controls every aspect of plant growth and development. The use of different phytohormones and PGRs during crop production and postharvest technologies has been considered as an emerging issue by many authors in recent years [9,11]. Brassinosteroids (BRs), as a new class of phytohormones, play roles in modulating plant growth and development [11,12,13,14,15]. There are little and contradictory reports regarding the effects of BRs on ethylene biosynthesis and signaling pathways especially in cut flowers. Most of the studies indicate the promoting roles of BRs on ethylene production and subsequent ripening and senescence processes in different horticultural crops [8,16,17,18,19,20]. Treatment of harvested tomato fruits with brassinolide (a synthetic brassinosteroid) resulted in the acceleration of fruit ripening, decrease in total chlorophylls, and increase in lycopene content, whereas brassinazole prevented chlorophyll degradation and lycopene accumulation, and delayed the ripening and senescence processes. The gene expression analysis showed that the expression of ACC synthase (ACS) genes including LeACS_2_ and LeACS_4_, as well as ACC oxidase (ACO) genes including LeACO_1_, LeACO_4_, and LePSY_1_ are increased in response to the brassinolide treatment. However, fruit treated with brassinazole showed the opposite effects. The authors suggested the involvement of BRs in the development of fruit quality attributes and ethylene-mediated fruit ripening in tomato [19].

Recently the dual and opposite effects of different levels of BRs on ethylene production in Arabidopsis plants has been reported and the possibility of such effects for different levels of BRs in other crops including the cut flowers should be considered [15]. On the other hand, by the increasing trends for restricting the use of chemicals in agricultural systems it is necessary to introduce safe and environmental friendly compounds for postharvest technology of different crops [11]. BRs, as natural compounds leave no chemical residues on the crop and environment and the possibility of using these phytochemicals should be illustrated. There is no study on the roles of BRs in the physiology of cut flowers including lisianthus. Therefore, this study was conducted to determine the effects of EBL, as a common BR, on ACC oxidase enzyme, senescence rate, quality, growth parameters, and vase life of lisianthus cut flowers. As a novel idea, we tried to use a wide range of BR concentration to see if it may decrease the activity of ACC oxidase at very low concentrations, as reported in Arabidopsis plants by Lv et al. [15], and extend the lisianthus cut flower vase life or not.

## 2. Results

### 2.1. ACO Enzyme Activity

**Figure 1:**
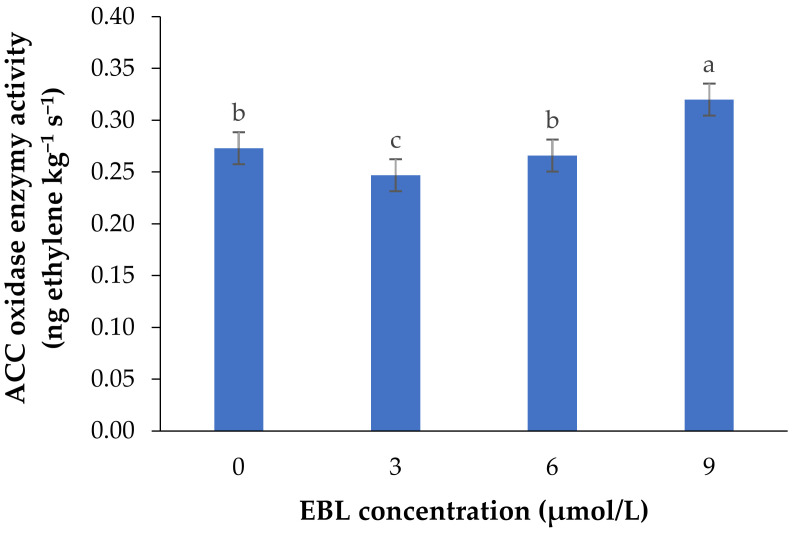
Mean data for the effect of EBL at different concentrations on ACC oxidase activity in lisianthus cut flowers during 14 days of storage at room temperature. Error bars represent the standard error of means of four replicates. Columns indicated by different letters are significantly different according to Duncan’s multiple range test (*p* ≤ 0.01).

The activity of 1-aminocyclopropane-1-carboxylic acid oxidase (ACO), as the final enzyme in ethylene biosynthesis converting ACC to ethylene, was significantly affected by the BR treatment (*p* ≤ 0.01) (Table 1 and Figure 1).

**Figure 2:**
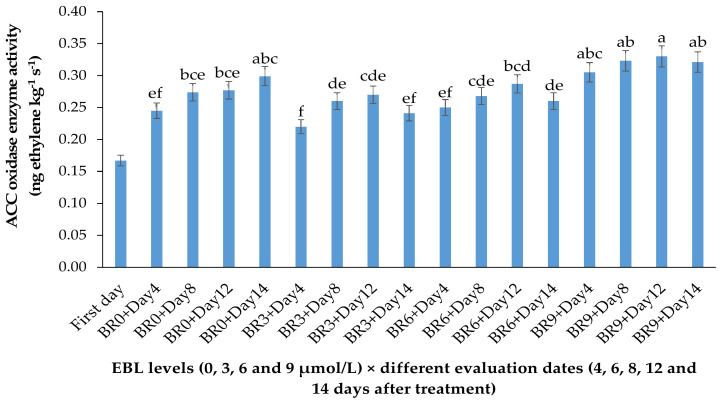
Effect of different concentrations of EBL on ACC oxidase activity of lisianthus cut flowers during 14 days of storage. Error bars represent the standard error of means of four replicates. Columns indicated by different letters are significantly different according to Duncan’s multiple range test (*p* ≤ 0.01). The first day is the harvest time of samples.

EBL at 3 µmol/L decreased the enzyme activity during the vase period, while with an increase in BR level the activity of ACO enzyme was increased, so that the highest enzyme activity was recorded at 9 µmol/L. During the vase life period, the lowest enzyme activity was seen after 4 days in flowers treated with 3 µmol/L EBL (Figure 2), and with an increase in storage time the enzyme activity was increased but remained lower than the flowers treated with 6 and 9 µmol/L at the end of the vase life period. While in flowers treated with 9 µmol/L EBL the difference between the first days and the end of storage was not significant (*p* ≤ 0.01).

### 2.2. Chlorophyll a and b Content

**Figure 3:**
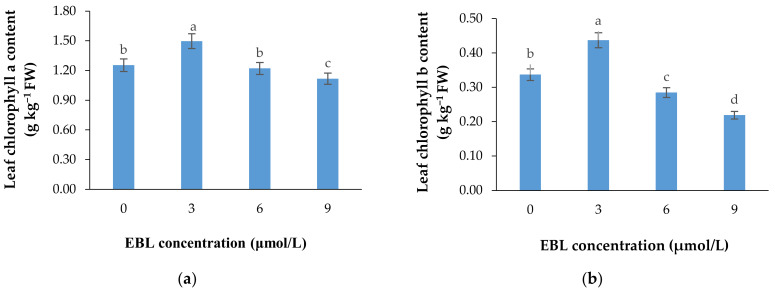
Mean data for the effect of EBL at different concentrations on chlorophylls a (**a**) and b (**b**) content of lisianthus cut flower leaves. Error bars represent the standard error of means of four replicates. Columns indicated by different letters are significantly different according to Duncan’s multiple range test (*p* ≤ 0.01).

**Figure 4:**
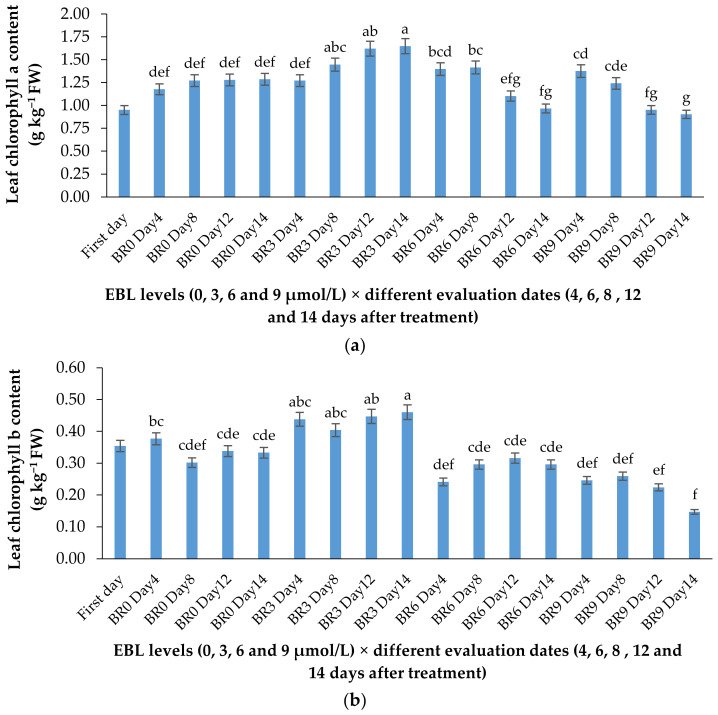
Effect of different concentrations of EBL on chlorophylls a (**a**) and b (**b**) content of lisianthus flower leaves at different evaluation dates. Error bars represent the standard error of means of four replicates. Columns indicated by different letters are significantly different according to Duncan’s multiple range test (*p* ≤ 0.01). The first day is the harvest time of samples.

According to Table 1 and Figure 3a,b, the lower concentration of BR not only was effective in retaining the chlorophyll content of the harvested flowers (Table 1), but also substantially enhanced the de novo biosynthesis of these valuable pigments vase period. EBL at 3 µmol/L significantly (*p* ≤ 0.01) enhanced both chlorophylls (a and b) biosynthesis in the leaves of the flowers during the vase period but decreased it at 6 and 9 µmol/L (Figure 3a,b). Until the end of the experiment, the chlorophyll biosynthesis continued to increase in control flowers but the increase in chlorophyll content of the flowers that received 3 µmol/L EBL was higher than all the flowers, indicating the role of lower BR levels in enhancing the chlorophyll biosynthesis. While EBL at 9 µmol/L decreased the chlorophyll content of the leaves during the vase period (Figure 4a,b).

### 2.3. Anthocyanin Content

**Figure 5:**
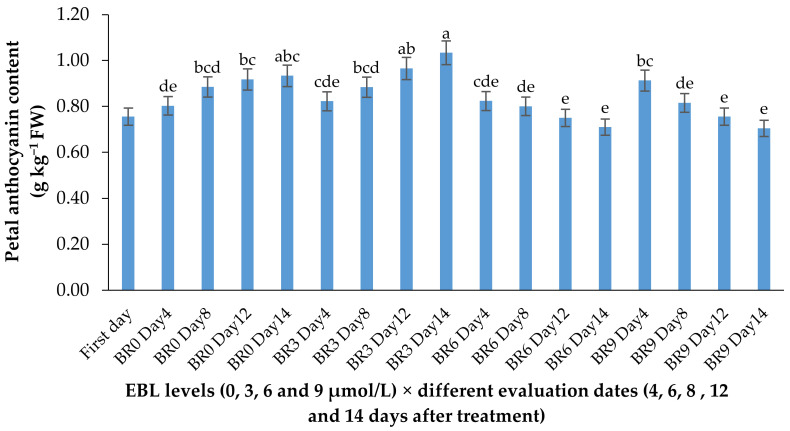
Effect of EBL on anthocyanin content of lisianthus flowers at different evaluation dates. Error bars represent the standard error of means of four replicates. Columns indicated by different letters are significantly different according to Duncan’s multiple range test (*p* ≤ 0.01). The first day is the harvest time of samples.

The anthocyanin content showed a slight increase in control flowers during the vase life period but showed no significant increase in flowers treated with higher levels of EBL. The increase in anthocyanin content of flowers treated with 3 µmol/L EBL was significantly (*p* ≤ 0.01) higher than 6 and 9 µmol/L (Table 1 and Figure 5).

### 2.4. MDA Production Rate

**Figure 6:**
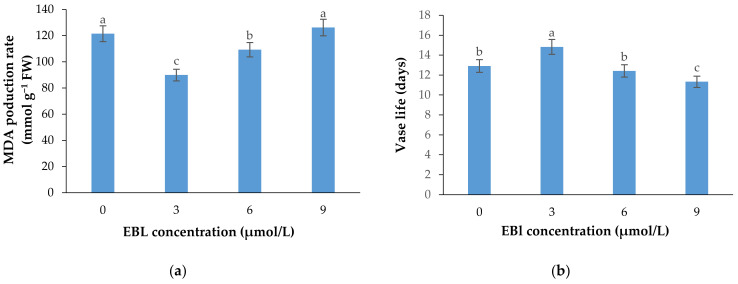
Mean data for the effects of EBL at different concentrations on MDA production rate (**a**) and vase life (**b**) of lisianthus cut flowers. Error bars represent the standard error of means of four replicates. Columns indicated by different letters are significantly different according to Duncan’s multiple range test (*p* ≤ 0.01).

MDA production, as an important indicator of cell membrane integrity status and senescence rate, was significantly (*p* ≤ 0.01) affected by the EBL treatment (Table 1 and Figure 6a). The MDA production in plants exposed to EBL exhibited different patterns. The MDA production substantially decreased at 3 µmol/L concentration. The MDA production was increased gradually with the increasing level of EBL, and reached the maximum at 9 µmol/L concentration. There was no significant difference between 9 μmol/L EBL and control.

### 2.5. Flower Vase Life

Flowers treated with 3 µmol/L EBL showed a significantly (*p* ≤ 0.01) higher vase life than the control and the flowers treated with 6 and 9 µmol/L EBL (Table 1 and Figure 6b). The vase life of flowers treated with 9 µmol/L EBL was significantly (*p* ≤ 0.01) lower than the control, indicating the adverse effects of higher BR concentrations on cell senescence rate and flower vase life.

### 2.6. Vase Solution Uptake Rate (VSU)

**Figure 7:**
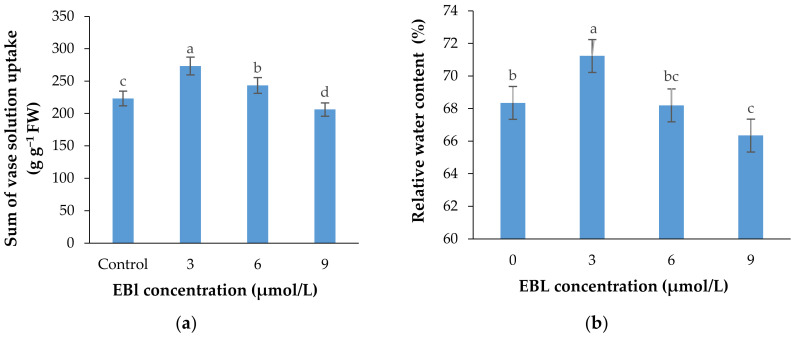
Sum of the vase solution absorbed by each lisianthus cut flower (mean of four replicates) (**a**) and the relative water content of the flowers (**b**) as affected by the EBL treatment at different concentrations. Error bars represent the standard error of means of four replicates. Columns indicated by different letters are significantly different according to Duncan’s multiple range test (*p* ≤ 0.01).

**Figure 8:**
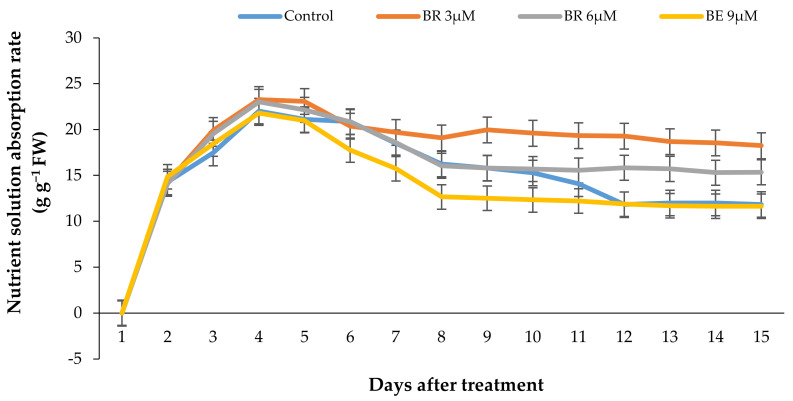
Effect of different EBL concentrations on the vase solution absorption pattern in lisianthus cut flowers in response to the EBL treatment during 15 days of keeping at room temperature. Error bars represent the standard error of means of four replicates.

The rate of vase solution absorption by the flowers was significantly affected with the EBL treatment (*p* ≤ 0.01). Similar to other parameters, EBL at 3 µmol/L significantly (*p* ≤ 0.01) increased the ability of the stems in absorbing the nutrient solution and with an increase in BR concentration the VSR rate was decreased in flowers (Figure 7a and Figure 8). Figure 7a shows that the total vase solution absorbed by the flowers treated with 3 µmol/L EBL was substantially higher than other flowers and the lowest value belongs to 9 µmol/L.

The pattern of VSU rate by the flowers in response to different treatments has been shown in Figure 8. According to Figure 8, the nutrient solution uptake rate showed a rapid increase in all experimental units during the first days and decreased rapidly in all the flowers after 5 days, except the flowers treated with 3 µmol/L EBL, which showed a slight decrease in VSU and remained constant until the end of the vase life period.

### 2.7. Relative Water Content

The relative water content of the flowers was significantly (*p* ≤ 0.01) affected by the treatments (Table 1, Figure 7b). The highest and lowest relative water contents were recorded in flowers treated with 3 and 9 µmol/L EBL, respectively. The difference between the control and 6 µmol/L EBL was not statistically different.

## 3. Discussion

Different concentrations of EBL showed opposite effects on all the evaluated parameters. EBL at 3 µmol/L EBL significantly decreased the activity of (ethylene-forming enzyme) EFE, enhanced different physiological parameters of the flowers including water uptake rate, chlorophyll and anthocyanins contents, and decreased the MDA production rate resulting in a significant increase in the flower vase life. While, at 6 µmol/L it had no significant effects and at 9 µmol/L it had a significantly adverse effect on flower physiological parameters. It seems that EBL affects the physiology of the cut flower mainly by affecting ethylene production via modulating the activity of ethylene biosynthesis pathway. The phytohormone ethylene plays the central roles in tissue senescence via activating different genes for degrading enzymes [8]. By converting S-adenosyl-Met (SAM) to ACC, ACS provides the precursor for ACC oxidase which converts ACC to ethylene and HCN by ACO in an oxygen-dependent process. Although the conversion of SAM to ACC has been considered as the rate-limiting step in ethylene biosynthesis pathway, however, recent studies show that in some cases ACC oxidase also plays an important role in the regulation of ethylene biosynthesis pathway [15,21]. In this study, EBL at a low concentration significantly decreased the senescence rate and enhanced the vase life of lisianthus cut flowers by decreasing the ACO activity and ethylene production and enhancing chlorophyll biosynthesis, anthocyanin accumulation, and vase solution absorption rate. Ethylene is the key signal activating different genes for degrading enzymes and senescence progress and it seems that very low levels of BR by decreasing ethylene production delay the senescence related events and extend the flower vase life.

Membrane deterioration is an early and characteristic feature of cut flowers irreversible senescence. An increased in lipid peroxidation caused phospholipid-degrading enzymes, such as phospholipase D and lipoxygenase, resulting in a loss of membrane integrity, which has been noted in the senescing flower tissues. Flower senescence is accompanied with increased permeability of flower cells and increased free radicals and ROS production, in which the activation and production of them are mainly modulated by ethylene [5]. The MDA production rate is an indicator of cell membrane integrity and senescence rate, and the levels of MDA produced by the cells show the deterioration rate of the cell membranes [6,8,9]. It seems that the role of EBL in decreasing ethylene production is the main reason for providing cell membrane integrity resulting in decreased MDA production. In addition, the flowers treated with lower levels of exogenous BR showed a significantly higher chlorophyll content and water absorption rate. Interestingly, these flowers had a higher anthocyanin content making them more attractive and marketable. While BR at higher levels had a negative effect on flower vase life due to enhancing the ACC oxidase activity and ethylene production leading to the activation of lipases, phospholipases, peroxidases, and lipoxygenases, which are responsible for membrane deterioration and enhanced MDA production rates [8]. In addition, chlorophyllases are activated by ethylene resulting in chlorophyll degradation [22]. With an increase in EBL concentration, the adverse effects of the phytohormone on the vase life of flowers were increased, demonstrating the promoting effects of higher BRs levels on senescence via enhancing the ACC oxidase activity and ethylene production rate leading to the activation of degrading enzymes. According to the reports of Lv et al. [15], BRs at very low levels may inhibit ethylene biosynthesis in Arabidopsis plants via modulating the brassinosteroid-regulated transcription factors (BES1 and BZR1), but at high concentrations, enhance ethylene biosynthesis by modulating the activity of ethylene biosynthesis enzymes and influencing auxin signaling for increasing ethylene production.

The higher relative water content of the flowers and the higher vase solution absorption rate in the flowers treated with the lower BR level (Figure 7b) indicate that this treatment has a promotive effect on the growth parameters of the flower resulting in a higher capacity for water uptake. While the higher EBL concentration decreases the water uptake rate possibly by enhancing a series of growth inhibitory signals including ethylene and ABA.

For the first time, our results demonstrate that the higher levels of BRs may enhance the ethylene biosynthesis via enhancing the ACC oxidase activity in cut flowers and harvest crops, while at very low levels decrease ethylene production and related senescence processes. The results of this study also explain, to some extent, the differences and in some cases the contradictory reports regarding the effects of BRs in enhancing or decreasing ethylene production and related consequences on ripening and senescence in some crops. It seems that very low differences in BRs concentrations may result in an opposite effect, so that low levels will decrease ACS and ACC oxidase gene expression and enzyme activity, while higher concentrations will probably enhance them.

Another interesting result obtained in this study was the positive effects of lower BR concentration on chlorophyll and anthocyanin biosynthesis and accumulation in lisianthus cut flowers. The biosynthesis of chlorophylls is very important for providing the possibility of photosynthesis and subsequent carbohydrate production, which is very important for the survival of the plants and cut flowers. While elevated levels of ethylene produced by higher BR levels is the cause for the expression of chlorophyllases genes and activity of related enzymes resulting in decreased chlorophyll content in the leaves [22], the modulating effects of BRs on chlorophyll biosynthesis and retention maybe due to the modulation of auxins and cytokinins signaling pathways [23,24].

Anthocyanins, derived from phenolics in the last steps of phenylpropanoid pathway, are among the most important color pigments in most of the cut flowers including lisianthus, determining the attractiveness and marketability of the flower [2]. Our results indicate the positive effects of lower EBL concentration on anthocyanin biosynthesis or accumulation. BRs have been shown to enhance the biosynthesis of different phenolics and anthocyanins in some fruits by upregulating the genes involved in phenylpropanoid pathway such as phenylalanine ammonialyase and chalcone synthase [11,17,25,26]. In grape berries, BR increased the transcript levels of F3H, F3′H, F3′5′H, UFGT, and regulatory MYBA1 genes involved in anthocyanin biosynthesis pathway, indicating that BRs directly magnify the phenolics and anthocyanins biosynthesis pathways [17]. In addition, these phytohormones have been reported to indirectly enhance the biosynthesis of anthocyanins via upregulating the cytokinin signaling pathway in Arabidopsis plants [27], and our results indicate that BRs are involved in anthocyanin formation in lisianthus cut flowers, in which the possible mechanisms remain to be illustrated by future studies.

## 4. Materials and Methods

### 4.1. Cut Flower Preparation and Treatments

The flowers of lisianthus (*Eustoma Grandiflorum* cv. Echo) were harvested in the morning when they had three fully opened flowers in the inflorescence from a commercial lisianthus growing greenhouse located in Pakdasht (Karaj, Iran), and transferred to the post-harvest physiology laboratory of Urmia University. All the flowers were of the same size and height. The base of the stems was put in the water during transport. The stems were recut under distilled water with a sterile sharp knife to decrease the height of the stems to 40 cm and one third of the lower leaves were removed. For each treatment, four sheaf’s of lisianthus flowers, with five inflorescence in every sheaf, were transferred to a 600 mL glass jar with 400 mL of distilled water containing only 2% sucrose.

### 4.2. Treatment of the Flowers with EBL

Pure EBL as a common BR was prepared from Sigma-Aldrich (USA). To prepare the EBL solutions (0, 3, 6, and 9 μmol/L), the appropriate amount of EBL to reach the desired concentration was dissolved in ethanol and then made to volume with distilled water. The final concentration of ethanol used for dissolving the EBL was about 0.3% (3 mL ethanol in 1000 mL of EBL solution), and the same concentration of ethanol was added to distilled water to treat the control flowers. The flowers were sprayed with 30 mL of the solution. Each treatment was performed on four plants (cut flowers), and two observations from two different parts of the flower were made for each experimental unit. The experimental units (treated and control flowers) were kept in a room with an average temperature of 25 °C and 70% RH under a lightening regime consisting of 13 h of light and 11 h of darkness. The required light was provided with 4 fluorescent lamps consisting of yellow and white lights. ACO (1-aminocyclopropane-1-carboxylic acid oxidase) enzyme activity, chlorophyll and anthocyanin contents of the leaves and petals, malondialdehyde (MDA) production rate of the petals, water absorption rate of the stems, and the vase life of the flowers were determined after 4, 8, 12, and 14 days of the vase period.

### 4.3. ACC Oxidase Activity Assay

The activity of ACC oxidase enzyme was determined using the method described by Fernández-Maculet and Yang [28] with a slight modification. One gram of the petal tissue was added to the extraction medium containing 0.1 mol Tris (pH 7.2), 10% (*w*/*v*) glycerol, 3 mmol/L sodium ascorbate, and 5% (*w*/*v*) PVPP and rubbed. After the slurry melted, it was filtered through a cheesecloth and centrifuged at 10,000× *g* for 35 min. The supernatant (crude extract) was used for the enzyme assays. The enzyme activity was assayed at 30 °C in 2 mL reaction mixtures containing a 1.7 mL extraction buffer (without PVPP), 50 μmol/L FeSO_4_, 1 mmol/L ACC, and 0.2 mL crude extract. Ethylene produced in the head space of 10 mL capped tubes after 1 h incubation was determined by GC (gas chromatography). For in vivo assays, 0.6–0.8 g of the flower tissue were first incubated with 10 mmol/L ACC and 0.4 mol mannitol for 1 h; the vials were then sealed and the ethylene produced during the following 1 h was measured.

### 4.4. Chlorophyll Content Evaluation

Chlorophylls a and b contents were determined using the method described by Arnon [29]. Chlorophyll extraction was done with a mixture of acetone and water at a ratio of 80:20 (*v*/*v*). In addition, 100 mg of leaf tissue was homogenized with 5 mL of 80% acetone solution, using a laboratory blender for 2 min and transferred to a micro tube to be centrifuged at 6000× *g* for 10 min. The supernatant was used for chlorophyll assay and putted in a 50 mL tube covered with aluminum foil to avoid oxidation of chlorophyll from light. Absorption was measured at 663 and 645 nm using a spectrophotometer. Chlorophyll contents were calculated according to the following formula and expressed as mg g-1 fresh weight.

Chlorophyll a (g kg^−1^ F.W) = (19.3 × A663 − 0.86 × A645) × X/100 (1)

Chlorophyll b (g kg^−1^ F.W) = (19.3 × A645 − 3.6 × A663) × X/100 (2)

where A645 = absorption value at 645 nm, A663 = absorption value at 663 nm, Χ = total volume of filtrate.

### 4.5. Determination of Anthocyanin Content

The anthocyanin content of the petals was determined using the method described by Wagner [30] with a slight modification. In addition, 100 mg of the petal tissue was soaked in 5 mL acidified methanol (containing 99% ethanol and 1% HCl). The tissue was crushed and kept for 24 h at 25 °C in a dark room. Finally, 5 mL of ethanol was added to the extract and the solution was then centrifuged at 4000× *g* for 10 min and kept at 4 °C. The supernatant was used for the anthocyanin assay. The absorption rates at 530 and 657 nmol/L were determined using a spectrophotometer and the anthocyanin content was calculated.

### 4.6. MDA Production Rate

The MDA rate as an indicator of lipid peroxidation and cell senescence rate was measured according to the method described by Heath and Packer [31]. In addition, 200 mg of petal sample was homogenized in 5 mL of 0.1% trichloro acetic acid (TCA). The homogenate was centrifuged at 10,000× *g* for 5 min. Then, 4 mL of 0.5% TCA + 1 mL of 5% thiobarbituric acid (TBA) were added to the solution and kept at 95 °C for 30 min and then quickly cooled in an ice bath. After centrifugation at 10,000× *g* for 10 min, the absorbance of the supernatant was recorded at 155, 532, and 600 nm. The MDA content was calculated using the following Equation (3):

MDA (mmol g^−1^ FW) = (A532 − A600/155) × 1000 (3)

### 4.7. Determining the Flower Vase Life

The average vase life of the cut flowers was counted from the day of transferring the flowers to the solutions, and was assessed to be terminated when the flowers lost their 50% of ornamental/display value (showed color change and wilting) [32]. The flowers were examined on a daily basis and symptoms such as wilting of petals, bending of the neck of the flower, leaf curvature, and burning of the petals edge were considered in determining the vase life. Florets with unfolded petals on stems, which had not bent, were considered as open florets.

### 4.8. Vase Solution Uptake Rate (VSU)

The VSU rate was determined according to Damunupola [33]. Weights of the vases containing vase solutions without the cut spikes were recorded daily during the vase life evaluation period. The average daily VSU rate was calculated by the formula:

NSA rate (g g^−1^ initial fresh weight (IFW) = (St^−1^ − S^t^)/IFW of the stem (4)

where St is the weight of the pot solution (g) at t = evaluation day and St^−1^ is the weight of the pot solution (g) on the previous day.

### 4.9. Relative Water Content

The relative water content of the flowers was measured according to González and González-Vilar [34]. Leaf discs of the flower spikes were harvested from at least three leaflets and immediately weighed to obtain fresh weight. They were subsequently immersed in deionized water overnight at 4 °C. The next day, leaf discs were reweighed after blotting to dryness and then were put in a dryer to obtain the dry weight. The relative water content was obtained as follows:

RWC = (FW − DW)/ (SW − DR) × 100(5)

where RWC = relative water content, FW = fresh weight, DW = dry weight, SW = water saturated weight.

### 4.10. Statistical Analysis

The experiment was carried out as a completely randomized design with EBL at 4 levels (0, 3, 6, and 9 µmol/L) and 4 evaluation dates (4, 8, 12, and 14 days after treatment and the beginning of storage) and four replications with five observations for every replication. All statistical analyses were performed according to the general linear model procedure of the statistical analysis system (SAS, version 0.2) and mean data comparisons were performed by Duncan’s multiple range test. Differences at *p* ≤ 0.01 were considered as significant.

## 5. Conclusions

From the results of this study, we can conclude that EBL as a common brassinosteroid modulates the physiological responses of lisianthus cut flowers resulting in an enhanced or decreased vase life depending on the concentration used. Our results strongly suggest that the effect of this natural and environmental friendly compound on different aspects of the cut flower is highly dose dependent, so that at a very low concentration decreases the rate of senescence and deteriorative process mainly due to decreasing the ethylene production, at least by decreasing the ACC oxidase activity, while at higher concentrations leaves opposite effects resulting in the decreased vase life. Due to the positive effects of this phytohormone at low concentrations, in future studies, it is better to evaluate lower concentrations, as well (0 < EBL < 3 µmol/L). Since BRs as bioactive compounds play crucial roles in different signaling pathways of the plant cells, more detailed future studies with a wide range of brassinosteroids levels will illustrate the role of these compounds on the gene expression and enzyme activity of other enzymes in the ethylene biosynthesis pathway. Moreover, interactions of BRs with other phytohormones are important for determining the postharvest behavior of cut flowers and may be an important subject for future studies.

## Figures and Tables

**Table 1:** One-way ANOVA for the effect of 24-epibrassinolide (EBL) and vase life duration (VLD) on ACC oxidase activity, chlorophylls (Ch) content, anthocyanin content, MDA production rate, vase life, and relative water content of lisianthus cut flowers.

	Mean Squares						
	ACC Oxidase Activity (ng ethylene kg^−1^ s^−1^)	Chlorophyll a (g kg^−1^ FW)	Chlorophyll b (g kg^−1^ FW)	Anthocyanin Content (g kg^−1^ FW)	MDA Production Rate (mmol g^−1^ FW)	Vase Life (Days)	Relative Water Content (%)
EBL	0.350 **	0.727 **	0.215 **	0.054 **	4962.530 **	116.27 **	102.848 **
Vase life duration	0.070 **	0.19 **	0.008 **	0.002 *^ns^*	2836.572 **	0.727 **	1115.706 **
EBL × VLD	0.018 *^ns^*	0.21 **	0.015 **	0.027 **	236.571 *^ns^*	0.16 **	15.85 **
Error	0.015	0.016	0.002	0.009	145.092	0.29	23.19

** and *ns* represents significance at the *p* ≤ 0.01 level and non-significance according to Duncan’s multiple range test, respectively.

## Data Availability

The data presented in this study are available on request from the corresponding authors.
